# Perceptions on Facial Profile Aesthetics: A Survey of Young People and Orthodontists

**DOI:** 10.4317/jced.62939

**Published:** 2025-08-01

**Authors:** Beatriz Larena-Morencos, Ana Belén Macías-Gago, Iván Nieto-Sánchez, Víctor Gómez-Clemente

**Affiliations:** 1 Private Practice. Madrid; 2 Orthodontic Graduate Lecturer. Centro Odontológico Hospital San Rafael. University Francisco de Vitoria

## Abstract

**Background:**

The aim of this study is to understand the perceptions of young non-dental professionals and orthodontists on the aesthetics of facial profile.

**Material and Methods:**

Perceptions were assessed using seven types of profile for both sexes, which represented different relations of the maxilla and mandible. In total, 50 young laypeople and 20 orthodontists were randomly selected to evaluate the aesthetics of each profile using numbers 1 to 10 to rank them in order of attractiveness. Aesthetics score was considered as a quantitative variable and comparison between groups was done with an ANOVA, and age and orthodontic treatment history of the evaluators from the young non-dental population was used as covariates to assess its possible effect.

**Results:**

The following female profiles were rated as most aesthetic: protruding jaw (*p*<.001; R2:21.9%) and retruded jaw (*p*<.001; R2:22.1%). The following male profiles were rated as most aesthetic: retruded maxilla (*p*=.001; R2:14.7%), bi-protruding maxilla and mandible (*p*<.001; R2: 54.4%) and straight maxilla and mandible (*p*<.05; R2:8.7%). Based on the sex of the evaluator, only significant differences were observed in these two male profiles. The straight profile showed significant differences (*p*<.10) with a moderate effect (4.3%), and the bi-retruded profile (*p*<.05) also had a moderate effect (8.5%); these were rated higher by men. Insufficient statistical evidence was found to admit that a history of orthodontic treatment influences aesthetic perception of male or female profiles. For all the variables, evaluators from the young population scored higher than orthodontists for both male and female profiles.

**Conclusions:**

The straight profile was considered more aesthetic for both sexes. Differences in aesthetic perception depending on sex and previous clinical history of orthodontic treatment were minimal. It can be concluded that the young lay population is less demanding in its evaluations than orthodontists.

** Key words:**Orthodontics, facial profile, aesthetic, aesthetic perception.

## Introduction

The objective of orthodontic treatment is to achieve an adequate and functional occlusion combined with a balanced and aesthetically pleasing facial appearance.

Numerous methods are used to measure the aesthetics of the smile, face and facial profile; most of them are based on measurements related to symmetry and average features among individuals [1]. However, beauty standards and aesthetic preferences of the population are influenced by sociocultural factors and can change as society does. An example is the evolution of aesthetic preferences of Western culture towards a profile with more protruded and thick lips, as well as a more prominent chin that Pogrel [2] observed throughout the 20th century.

On the other hand, aesthetic improvement is a powerful motivating factor that leads both adult and children/adolescent patients to seek orthodontic treatment [3,4]. Despite the fact that the main motivation to get an orthodontic treatment is facial aesthetics, very few patients are aware of how their profiles are and that they can be modified with orthodontic treatment; in fact, only half of the population is aware of their profile, and they tend to give their own profiles higher scores than those given by an orthodontist [5,6].

To date, numerous studies have been conducted on the aesthetics of facial profile and aesthetic perception among non-professional populations. For some professionals, achieving the desired aesthetic standard for a patient is a difficult task because of the subjective nature of facial aesthetics evaluation and perception, according to studies conducted by Kumar *et al*. [7] and Cochrane *et al*. [8]; however, most studies [9-11] conclude that there are no significant differences between the aesthetic perceptions of professionals and the general population. Researchers also agree that orthodontists tend to be more sensitive and stricter in their judgment than the general population because of their training in and knowledge of malocclusions. In addition, they appear to have a greater ability to distinguish among profile changes, while the untrained observer tends to focus on other extrinsic facial characteristics, such as chin shape, nose size and shape, hair colour and style, etc., which can influence their perception of attractiveness [12]. In addition, the general population’s perception of facial profiles adapts to social norms and beauty culture—criteria which can change over time [13].

Therefore, it is argued that these criteria should be known by orthodontists and considered when developing a treatment plan to achieve greater patient satisfaction.

## Material and Methods

**Figure 1:**
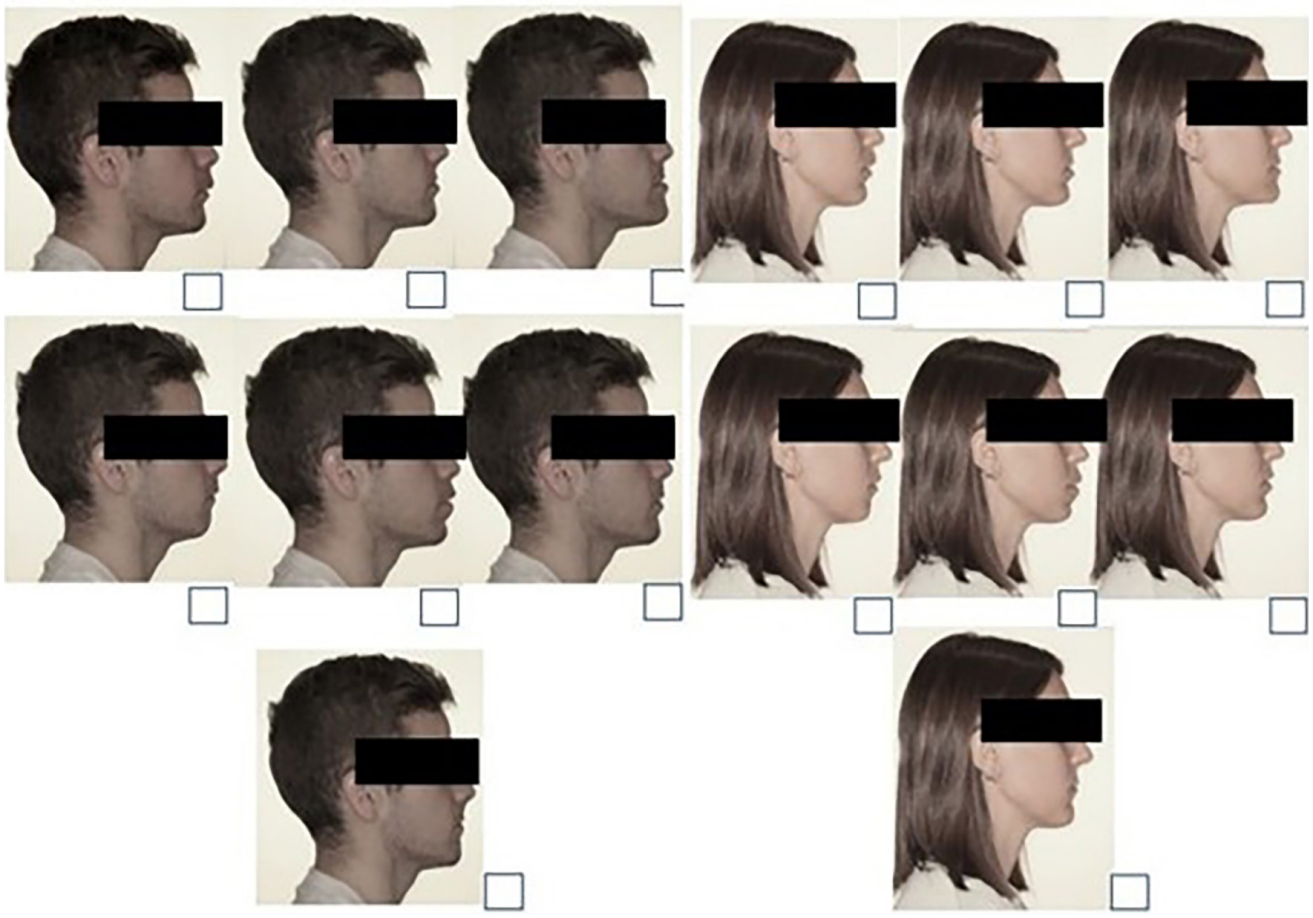
Male and Female images with different modification shown to the participants.

For this study, a survey was designed to evaluate perceptions on the aesthetics of facial profiles based on the scores given to different profiles. For this, two models were included—a male and a female—who were photographed in a natural head position. These photographs were digitally modified to create six new profiles: 1) prognathic maxilla, 2) retrusive maxilla, 3) prognathic mandible, 4) retrusive mandible, 5) bidentoalveolar protrusion, and 6) bidentoalveolar retrusion. The modifications were made using the online photo editor Pixlr. The position of the lips, soft point A of the maxilla and soft Pogonion point and soft point B at the mandibular level were modified. The photographs were modified to black and white to avoid the influence of parameters such as skin colour, eyes or hair on the aesthetic evaluation. In the survey, the evaluators had to score the profiles on a scale of 1 to 10, with 1 being the least aesthetic and 10 being the most aesthetic (Fig. 1).

To conduct the survey, young non-professionals in dentistry aged 18 to 30 years were selected; these included both postgraduate dental students and patients who attended the dental centre of the San Rafael University Hospital in Madrid. The study also included orthodontists with a specialty from any Spanish university and without an age limit. This was an unmasked study, so the researcher and the patients were aware from the beginning of the study objective and parameters that would be evaluated later with the photographs. For the young population unrelated to dentistry, their sex and history of orthodontic treatment were considered as possible variables that may affect their aesthetic perception of the facial profiles. Descriptive analyses of qualitative variables were carried out using counts and percentages, and for quantitative variables, it was performed through range, quartiles, mean and standard deviation. Aesthetic score is considered a quantitative variable. A comparison between the groups was carried out with an ANOVA, and the age and history of orthodontic treatment of the evaluators from the young population unrelated to dentistry were used as covariables to assess their possible effect.

## Results

Data from a total of 70 participants was collected: 20 orthodontists and 50 young people from the general population unrelated to orthodontics. Of the total population, 51.4% (36) were women, and the remaining 48.6% (34) were men. The age range was between 18 and 30 years in the young population group, and in the orthodontist group, it was 27 to 50 years. It is observed that in the young population group, the participation of men and women was balanced (27 and 23); while in the group of orthodontists, a clear majority of women was observed (65% vs 35%). The mean age of participants in the young group was almost 26 years, significantly lower than the mean age of participants in the professional group (i.e., 34 years), which was a statistically significant difference (*p*<.001).

**Figure 2:**
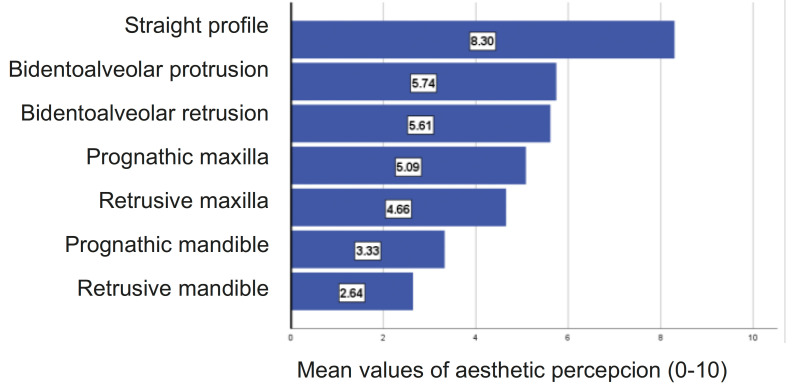
Percepcion of male facial aesthetics. N=70.

**Figure 3:**
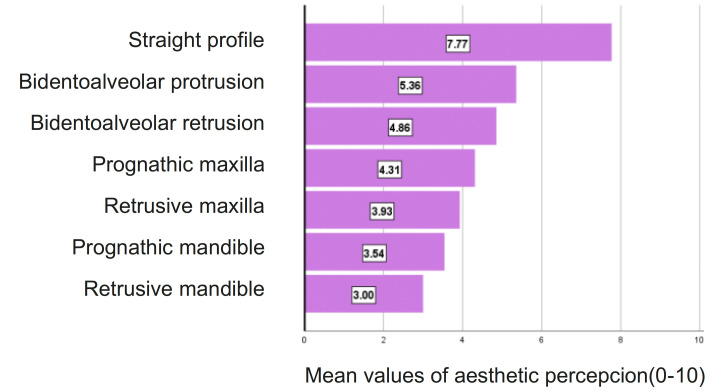
Percepcion of female facial aesthetics. N=70.

- Profile assessment 

1) Among the male profiles (Fig. 2), the one perceived as the most aesthetic was defined with a straight maxilla and mandible (8.30 points out of 10), followed by the one with dentoalveolar biprotrusion (5.74 points) and dentoalveolar biretrusion (5.61). The least aesthetic profile was the one with the prognathic mandible (2.64 points).

2) Among the female profiles (Fig. 3), the one perceived as the most aesthetic was also the one with a straight maxilla and mandible (7.77 points), followed by the one with dentoalveolar biretrusion (5.36 points). The least aesthetically pleasing profiles were the following three: retrusive maxilla (3.93 points), prognathic mandible (3.54) and dentoalveolar biprotrusion (3.00).

Aesthetic perception of the young population and orthodontists

Male profiles: In all the variables, it was observed that evaluators from the young population gave higher scores than the orthodontists; however, the differences were not statistically significant for all the profiles (at least *p*<.05; Fig. 1;  Table 1). Significant differences were only obtained for the following profiles.

• Prognathic maxilla: *p*<.001 and a large effect size (21.1%) 

• Retrusive maxilla: *p*<.01 and a moderate to high effect size (9.4%) 

• Dentoalveolar biprotrusion: *p*<.05 and a moderate effect size (8.1%) 

• Retrusive mandible: *p*<.05 and a moderate effect size (6.9%)

Female profiles: For most of the profiles, evaluators from the young population gave higher scores than the orthodontists. Significant differences (at least *p*<.05) appear in fewer variables than in male profiles and with smaller effect sizes ( Table 1) for the following profiles.

• Prognathic maxilla: *p*<.001 and a large effect size (16.8%) 

• Dentoalveolar biretrusion: *p*<.01 and a moderate effect (9.3%)

•In the retrusive mandible: *p*<.05 and a moderate effect (7.0%) 

As a general conclusion, we have statistical evidence proving that evaluators from the young population tend to always score the aesthetics higher as compared with orthodontists for any type of profile.

- Aesthetic perceptions according to the sex of the evaluator

When comparing the judgments given for male profiles, similar average values were observed for the scores by male evaluators and female evaluators, such that for 5 of the 7 variables, the differences did not reach statistical significance (*p*>.05): the straight profile almost reached significance (*p*<.10) with a moderate effect size (4.3%), while the dentoalveolar biretrusive profile reached significance (*p* <.05) with a moderate effect size (8.5%) but higher than the previous one. For both these profiles, men have given higher scores ( Table 2).

Very similar average values were observed between the judgments of women and men for female profiles. Therefore, there were no differences between genders that reached statistical significance (*p*>.05) nor any tendency towards significance (*p*>.10), which, together with very low and almost null effect sizes, lead us to conclude that the perception of female profiles does not depend on the sex of the evaluator ( Table 3).

In general, the overall conclusion is that the aesthetic perception of a profile does not depend on the sex of the evaluator, with some exceptions for male profiles.

Orthodontic treatment history

The results show that there were hardly any differences between an experience and no experience with orthodontic treatment. No significant differences (*p*>.05) were observed, nor were there effect sizes that reached at least a moderate level (except for a couple of exceptions). Therefore, enough statistical evidence has not been found to admit that a history of orthodontic treatment influences the evaluator’s aesthetic perceptions of profiles ( Table 4).

## Discussion

The straight profile was considered the most aesthetically pleasing for both men and women, and this view has been widely accepted in the literature [12,14-17].

The dentoalveolar biretrusive and dentoalveolar prognathic profiles were the second-best rated and considered accepTable for the male profile. For the female profile, only the dentoalveolar biretrusive one was considered accepTable, while the dentoalveolar prognathic profile was poorly rated. This result does not correspond with the literature studied, as in most studies, such as those by Türkkahraman [15] and Abu Arqoub [12], the dentoalveolar biretrusive and dentoalveolar prognathic profiles are among the best rated for both sexes. Perhaps our results for the female dentoalveolar prognathic profile have been worse than expected because of the digital modification of the photograph, which generated an unesthetic and disharmonious anatomy of the lips.

Regarding differences between the perceptions of a young, non-professional population and those of the orthodontist population, the results indicate that both groups have similar preferences for aesthetic profiles. However, the orthodontists are stricter in their ratings, while the general young population tolerates deviations from the norm better. In general, most studies on aesthetic perceptions between a non-professional population and orthodontists have obtained similar results [12,14-17]. Romani [18] has shown that in addition to orthodontists being stricter in their evaluations, they are also able to differentiate 1 mm of change in 50-70% of the profiles; while the non-professional population of dentistry is not able to spot these differences.

The literature has controversial results regarding differences in perception based on the sex of the evaluator. Many researchers, such as Oliveira [19], Todd [20], and Lü [21], did not find differences in the evaluation of the profile based on the sex of the evaluator. However, other researchers found such differences. Türkkahraman [15] observed that men rate convex female profiles more positively, while women rate concave profiles more positively. The differences found by Tole [14] were in terms of the level of scoring, with women generally offering lower scores. On the other hand, Cochrane [22-26] observed that women score the straight profile as more aesthetically pleasing than men, that is, they are more unanimous in their choice of the most aesthetically pleasing profile. Cala [17] observed statistically significant differences in scores based on sex, but the overall order of preferences for the profiles did not change. Their results are most similar to our study, as we only observed significant differences in the straight and dentoalveolar retrusive male profiles, for which men rated both the profiles more positively. However, in general, the same aesthetic preferences were maintained, therefore proving that the aesthetic perception of a profile does not depend on the sex of the evaluator.

Regarding the history of orthodontic treatment, it might be assumed that orthodontic patients are more familiar with aesthetics and the influence of malocclusions on the facial profile. However, in this study, no differences were observed between the scores of the young people who had received orthodontic treatment and those who had not, so it was not considered a significant variable. A few articles have studied the history of orthodontics as a possible variable; in those, the results are similar to those of this study [17]. Among them, the study by Arpino [23], which compares the aesthetic perception of patients undergoing orthognathic surgery, orthodontic patients and people who have not received orthodontic treatment, is worth noting. When divided into these categories, it was observed that the patients undergoing orthognathic surgery are more critical of the profile and less tolerant of deviations from the norm compared with orthodontic patients or those who have not received treatment. Milutinovic *et al*. [27] have also pointed out that small lower third and a full upper lip and high nasal tip contribute to a pleasing profile.

Finally, it is important to consider that some variables such as socio-economic level, ethnic background or the participant’s own facial profile may influence their aesthetic perceptions; these have not been taken into account in this study.

## Conclusions

• The straight profile for both men and women is considered the most aesthetically pleasing by both orthodontists and a young population .

• Orthodontists tend to be more demanding in their evaluations of facial profile aesthetics compared with a young, non-professional population.

• Hardly any differences were observed in the aesthetic evaluation based on the sex of the evaluator. The only differences were observed for the straight and biretruso male profiles, in which men rated both the profiles more positively than women.

• A clinical history of orthodontics is not a significant variable in influencing the perception of profile aesthetics.

## Figures and Tables

**Table 1:** Inferential Analysis. Contrast of the aesthetic perception of male and female profiles, based on the population to which the evaluator belongs.

MALE PROFILE	Mean (standard desviation)		Contrast test		Effect size: R2
	YOUNG POPULA. (n=50)	ORTHODON. (n=20)	Value	P-value	
Prognathic maxilla	5.68 (±1.94)	3.60 (±1.57)	18.14**	.000	.211
Retrusive maxilla	5.10 (±2.41)	3.55 (±1.57)	7.04**	.009	.094
Prognathic mandible	2.86 (±1.75)	2.10 (±1.37)	3.02	.087	.043
Retrusive mandible	3.64 (±2.02)	2.55 (±1.28)	5.01*	.029	.069
Bidentoalveolar protrusion	6.12 (±2.02)	4.80 (±2.09)	5.99*	.017	.081
Straight	8.34 (±1.12)	8.20 (±1.47)	0.19 NS	.668	.003
Bidentoalveolar retrusion	5.78 (±1.99)	5.20 (±1.96)	1.22 NS	.273	.018
FEMALE PROFILE	Mean (standard desviation)		Contrast test		Effect size: R2
	YOUNG POPULA. (n=50)	ORTHODON. (n=20)	Value	P-value	
Prognathic maxilla	5.42 (±2.05)	3.45 (±1.90)	13.70**	.000	.168
Retrusive maxilla	4.18 (±1.75)	3.30 (±1.52)	3.89	.053	.054
Prognathic mandible	3.70 (±1.85)	3.15 (±1.63)	1.34 NS	.251	.019
Retrusive mandible	4.62 (±1.86)	3.55 (±1.57)	5.13*	.027	.070
Bidentoalveolar protrusion	3.24 (±2.04)	2.40 (±1.76)	2.62 NS	.110	.037
Straight	7.60 (±1.74)	8.20 (±0.89)	2.14 NS	.148	.031
Bidentoalveolar retrusion	5.68 (±1.73)	4.55 (±1.28)	6.80**	.009	.093

N.S. = NOT significant † = Almost significant * = Significant ** = highly significant

**Table 2:** Inferential Analysis. Contrast of the aesthetic perception of male profiles, based on the gender of the evaluator. (N=70).

MALE PROFILE`S variables	Mean (standard desviation)		Contrast test		Effect size: R2
	MALE (n=34)	FEMALE (n=36)	Value	P-value	
Prognathic maxilla	5.06 (±1.91)	5.11 (±2.23)	0.01 NS	.916	.000
Retrusive maxilla	4.97 (±2.46)	4.36 (±2.14)	1.23 NS	.271	.018
Prognathic mandible	2.68 (±1.72)	2.61 (±1.66)	0.03 NS	.872	.000
Retrusive mandible	3.12 (±1.75)	3.53 (±2.02)	0.82 NS	.369	.012
Bidentoalveolar protrusion	5.44 (±2.40)	6.03 (±1.78)	1.36 NS	.248	.020
Straight	8.56 (±1.13)	8.06 (±1.26)	3.06	.085	.043
Bidentoalveolar retrusion	6.21 (±1.97)	5.06 (±1.87)	6.31 *	.014	.085

N.S. = NOT significant † = Almost significant * = Significant

**Table 3:** Inferential Analysis. Contrast of the aesthetic perception of female profiles, based on the gender of the evaluator. (N=70).

FEMALE PROFILE`S variables	Mean (standard desviation)		Contrast test		Effect size: R2
	MALE (n=34)	FEMALE (n=36)	Value	P-value	
Prognathic maxilla	5.15 (±2.31)	4.58 (±2.06)	1.16 NS	.285	.017
Retrusive maxilla	3.94 (±1.94)	3.92 (±1.52)	0.00 NS	.953	.000
Prognathic mandible	3.38 (±1.46)	3.69 (±2.08)	0.55 NS	.472	.008
Retrusive mandible	4.53 (±1.81)	4.11 (±1.86)	0.90 NS	.345	.013
Bidentoalveolar protrusion	2.71 (±1.66)	3.28 (±2.24)	1.46 NS	.231	.021
Straight	7.53 (±1.73)	8.00 (±1.37)	1.60 NS	.210	.023
Bidentoalveolar retrusion	5.53 (±1.62)	5.19 (±1.75)	0.69 NS	.410	.010

N.S. = NOT significant

**Table 4:** Table Inferential Analysis. Contrast of the aesthetic perception of male and female profiles, based on the population to which the evaluator belongs N=50 evaluators from young population.

MALE PROFILE	Mean (standard desviation)		Contrast test		Effect size: R2
	NO Ortodont. Treat. (n=19)	Orthodont. Treat. (n=31)	Value	P-value	
Prognathic maxilla	5.95 (±2.30)	5.52 (±1.71)	0.58 NS	.452	.012
Retrusive maxilla	4.95 (±2.20)	5.19 (±2.56)	0.12 NS	.730	.003
Prognathic mandible	2.84 (±1.68)	2.87 (±1.82)	0.00 NS	.956	.000
Retrusive mandible	3.47 (±2.34)	3.74 (±1.82)	0.20 NS	.653	.004
Bidentoalveolar protrusion	5.32 (±2.26)	6.61 (±1.71)	5.30 *	.026	.099
Straight	8.21 (±1.27)	8.42 (±1.02)	0.41 NS	.527	.008
Bidentoalveolar retrusion	5.74 (±2.26)	5.81 (±1.85)	0.01 NS	.906	.000
FEMALE PROFILE	Mean (standard deviation)		Contrast test		Effect size: R2
	NO Ortodont. Treat. (n=19)	Orthodont. Treat. (n=31)	Value	P-value	
Prognathic maxilla	5.74 (±2.62)	5.23 (±1.63)	0.73 NS	.398	.015
Retrusive maxilla	4.26 (±2.00)	4.13 (±1.61)	0.07 NS	.795	.001
Prognathic mandible	3.42 (±1.46)	3.87 (±2.06)	0.69 NS	.411	.014
Retrusive mandible	4.21 (±2.20)	4.87 (±1.61)	1.50 NS	.227	.030
Bidentoalveolar protrusion	3.16 (±2.43)	3.29 (±1.79)	0.05 NS	.826	.001
Straight	7.05 (±1.93)	7.94 (±1.55)	3.18	.081	.062
Bidentoalveolar retrusion	5.42 (±1.90)	5.84 (±1.64)	0.68 NS	.413	.014

N.S. = NOT significant † = Almost significant * = Significant

## Data Availability

The datasets used and/or analyzed during the current study are available from the corresponding author.
